# Assessing staining resistance of a CAD/CAM interpenetrating network composite material

**DOI:** 10.1186/s13005-018-0184-2

**Published:** 2018-11-26

**Authors:** Mehmet Mustafa Özarslan, Ulviye Şebnem Büyükkaplan, Çağatay Barutcigil, Merve Özarslan, Kubilay Barutcigil, Nurullah Türker

**Affiliations:** 1 Department of Prosthodontics, Akdeniz University, Faculty of Dentistry, 07 058 Antalya, Türkiye; 2 Department of Restorative Dentistry, Akdeniz University, Faculty of Dentistry, Antalya, Türkiye

**Keywords:** Interpenetrating network composite, IPN, Staining, ΔE_00_, CAD/CAM

## Abstract

**Background:**

Color change of dental materials over time because of staining agents has important effects on the long-term prognosis of dental esthetic treatments. In the present study, staining resistance of an interpenetrating network composite material with different translucency levels (translucent and high translucent) and various surface procedures for finishing were investigated.

**Methods:**

Same shade CAD/CAM interpenetrating network composite specimens having two different translucency levels were prepared using by a water cooling cutting saw. Specimens were grouped and different finishing procedures were performed. Then, specimens were kept in distilled water, red wine and coffee for different periods. Color was evaluated before and after exposure to staining liquids using a clinical spectrophotometer.

**Results:**

In the 7 days Glaze group (ΔE_00_ > 2.2), color difference was only perceivable in the specimens kept in red wine. In the 28 days red wine high translucent groups, only the high translucent Clinical group showed a clinically acceptable (ΔE_00_ < 2.2) color change. In the translucent groups kept in red wine, only the translucent Glaze group showed a clinically perceivable color change (ΔE_00_ > 2.2). In the 28 day measurements, all the translucent groups showed a clinically perceivable color change (ΔE_00_ > 2.2). Of the translucent groups kept in coffee for 28 days, it was observed that only the translucent Clinical group demonstrated a clinically non-perceivable color change (ΔE_00_ < 2.2).

**Conclusions:**

All the translucent groups kept in red wine showed a clinically perceivable color change after 28 days. Only the high translucent Clinical Kit group showed a clinically acceptable color change. Among high translucent and translucent specimens kept in coffee only the translucent Clinical group showed a clinically acceptable color change after 28 days.

## Background

Composites and ceramics are still the most prefered dental materials in restorative dentistry. However, the disadvantages such as brittleness and antagonistic natural tooth wear of dental ceramics and, easy staining and greater wear of composites have led researchers to overcome the deficiencies of these 2 conventional dental materials. Interpenetrating network composite (IPN) materials are quite new in materials in the dental market to cope with the above mentioned disadvantages of ceramics and composites [1, 2]. Traditional resin-based composites have an organic polymer matrix and inorganic filler particles [3, 4]. IPNs have a three-dimensional interconnected geometry. On the contrary to conventional composites that containing one continuous phase filled with inorganic particles, an IPN material contain 2 continuous interpenetrating networks [1], a polymer network (14%wt) together with a ceramic network (86%wt), and both of these materials fully interpenetrate each other [5]. Regarding to optical properties, IPN possess higher opacity originating from the shrinkage of the resin as a result of interfacial stress occurring between the ceramic framework and the polymer [6]. The selection of resin, the application of high pressure during the curing phase, and the silanization process enhanced bonding and helped overcoming the aesthetic problems by increasing the translucency of the material [6]. In the previous studies, it was shown that IPN possess lower hardness values [1, 7]. Thus wearing of the IPN material is easy and this phenomena causes significant reductions in surface gloss of finished material surfaces. A reduced gloss makes a surface more prone to staining. Progressively, as wear and roughness increase, the gloss decreases and the color changes [8–11].

It has been shown that composites undergo sorption of staining agents’ extrinsic pigments due to degradation of the organic matrix [10, 12–15]. Although IPN [polymer-infiltrated ceramic network (PICN)] materials have a resin matrix, most of their constituents are ceramic (86%). For this reason, staining and surface finishing properties can be different from traditional polymer and composite materials. Staining of contemporary composites has been widely investigated in in vitro studies. However, there is no report in the dental literature about the effects of different translucency levels on the staining resistance of IPN materials. Also, information about the effects of different finishing methods on the staining resistance of IPN materials is restricted since they are quite new generation materials in the dental market [16–19]. Therefore, the purpose of this in vitro study was investigation of the staining resistance of a high translucent and translucent IPN materials finished with various procedures then exposed to staining solutions.

## Methods

In the present study, CAD/CAM 2 M2 shade IPN blocks (Vita Zahnfabrik, Cuxhaven, Germany) were investigated. IPN blocks having two different translucency levels as “high translucent (HT) and translucent (T)” were selected for the present study. For each of two type of translucent groups, 90 specimens (totally 180) with dimensions of 12 × 14 × 2 mm were prepared using a water cooling and low-speed diamond saw (Isomed 1000, Buehler Ltd., Lake Bluff, IL, USA). The surface of the specimens were abraded with carborundum sandpaper. Specimens were cleaned ultrasonically and also with alcohol after grounding. After that all the specimens were dried with compressed air. HT and T groups specimens were divided into 4 groups. Different surface finishing procedures were applied to the specimens using the methods described in a previous study [20, 21]: Control group: no surface procedure applied; Technical group: Vita Enamic Technical Kit applied; Clinical group: Vita Enamic Clinical Kit applied; Glaze group: Vita Enamic glaze applied. After completing the surface procedures, the thickness of the each specimen was determined with a caliper (Mitutoyo Corporation, Tokyo, Japan). Specimens that had more than 0.05 mm thickness variation were excluded and replaced by a new specimen. All specimens were stored in an incubator at 37 °C for 24 h. Color change of the specimens was evaluated with distilled water, filtered coffee and red wine. Thus, 3 subgroups (a total of 24 subgroups, *n* = 15) for distilled water, coffee (Nestle, Vevey, Switzerland) and red wine (Doluca, Istanbul, Turkiye) were generated from each of the 8 groups (HT, T Control, HT,T Technical, HT, T Clinical, HT,T Glaze). Before the first measurement was performed, all the groups were exposed to the solutions in an incubator at 37 °C for 24 h. Coffee solutions were made up putting 15 g of coffee powder into 500 ml of boiled distilled water. After 10 min of stirring, coffee solutions were filtered through a filter paper. The color measurements of each of the specimens were measured by single investigator before immersion of the specimens in the solutions for 24 h, 7 and 28 days. For the prevention of plaque formation on the specimens, the solutions were changed in daily period. Color change was measured using by a clinical spectrophotometer (Vita Easyshade Advance, Vita Zahnfabrik, Cuxhaven, Germany). Each of the color change measurements was taken and recorded following washout of the specimens gently with distilled water for 5 min and drying with air. Calibration of the device was done before each color change determination performed. All measurements were made in single tooth mode. First on a white background (L* = 93.5, a* = 0.2, b* = − 1.5) and then on a black background (L* = 0, a* = 0, b* = 0) under the same lighting conditions (D65). All measurement results were recorded according to the CIELAB system. Shade differences between specimens were determined with a CIEDE2000 color system [22]. It was stated that color differences between two subjects could be calculated and it can be used for clinical evaluation using the CIELAB color system. But, the system has some limitations such as representing the hue values [23]. Thus, various CIELAB based color differences formulas were introduced to overcome the limitations of the system [24]. A recent research suggests that CIEDE2000 can be used for better evaluation of color differences [25]. This formula incorporates specific corrections for nonuniformity of CIELAB color space especially for the interaction between chroma and hue differences in the blue region, and a modification of a* coordinate of CIELAB, which mainly affects colors with low chroma [26]. Shade differences were transformed to CIEDE2000 using the formula previously described [27] and recorded as ΔE00.

In a previous study, the clinically acceptable threshold of color difference value was reported as 2.2 (ΔE_00_) [28]. This means that a ΔE_00_ value greater than 2.2 was a perceivable color change by an observer. Thus, in the present study ΔE_00_ > 2.2 was accepted for perceivable ΔE_00_ threshold. For the statistical analysis, normality distribution was evaluated to determine the color changes. First, a Kolmogorov-Smirnov test for normality was carried out. The result was below 0.05 (*p* < 0.05). Thus, non-parametric tests were performed for color change determination. The differences between the groups were analyzed with Kruskal–Wallis and Mann–Whitney U tests. Time-dependent variables were analyzed with Friedman and Wilcoxon tests. The statistical significance level was set as *p* < 0.05.

## Results

**Fig. 1:**
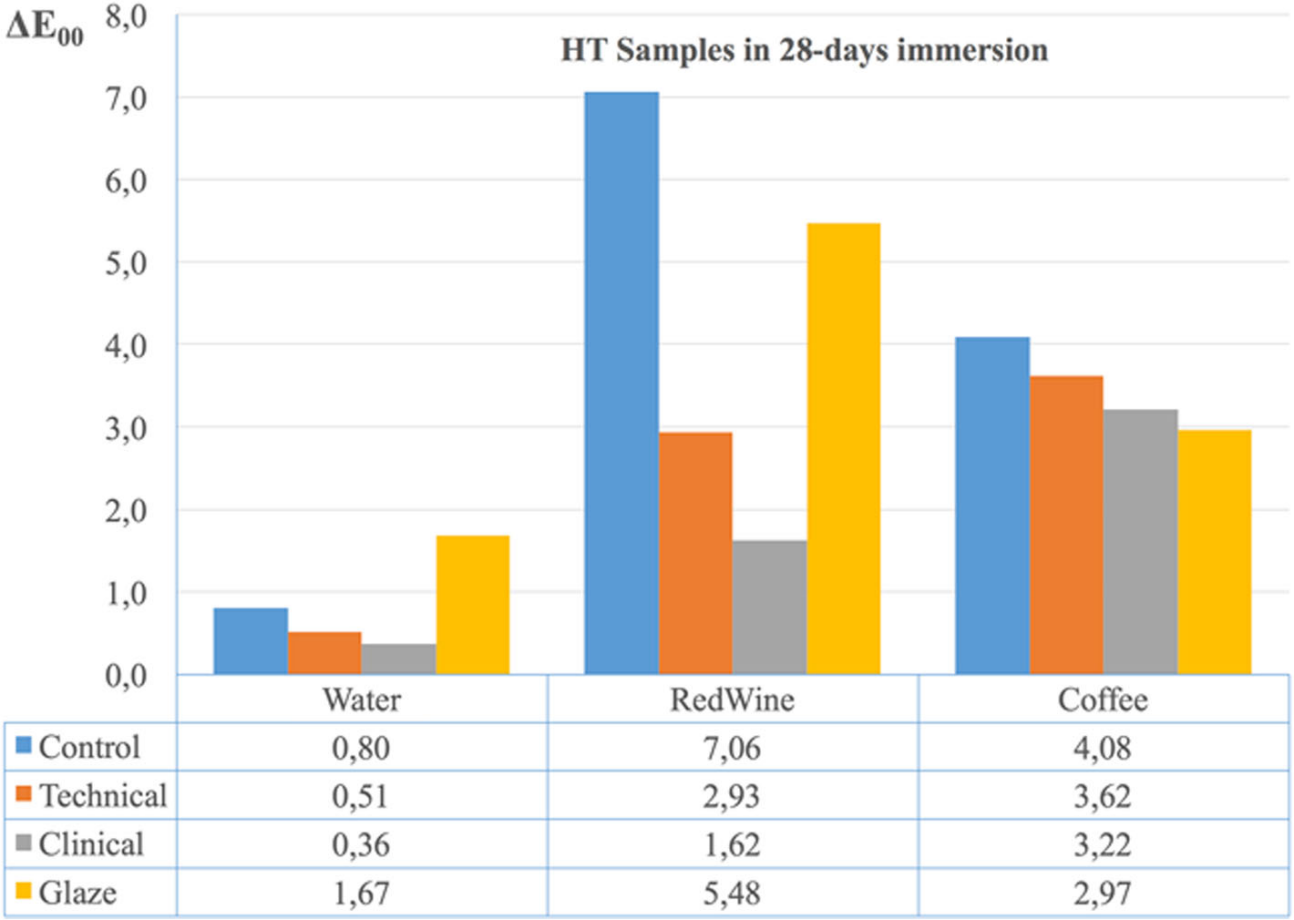
Color changes of high translucent polymer infiltrated ceramic network material after 28 days immersion in different drinks

**Fig. 2:**
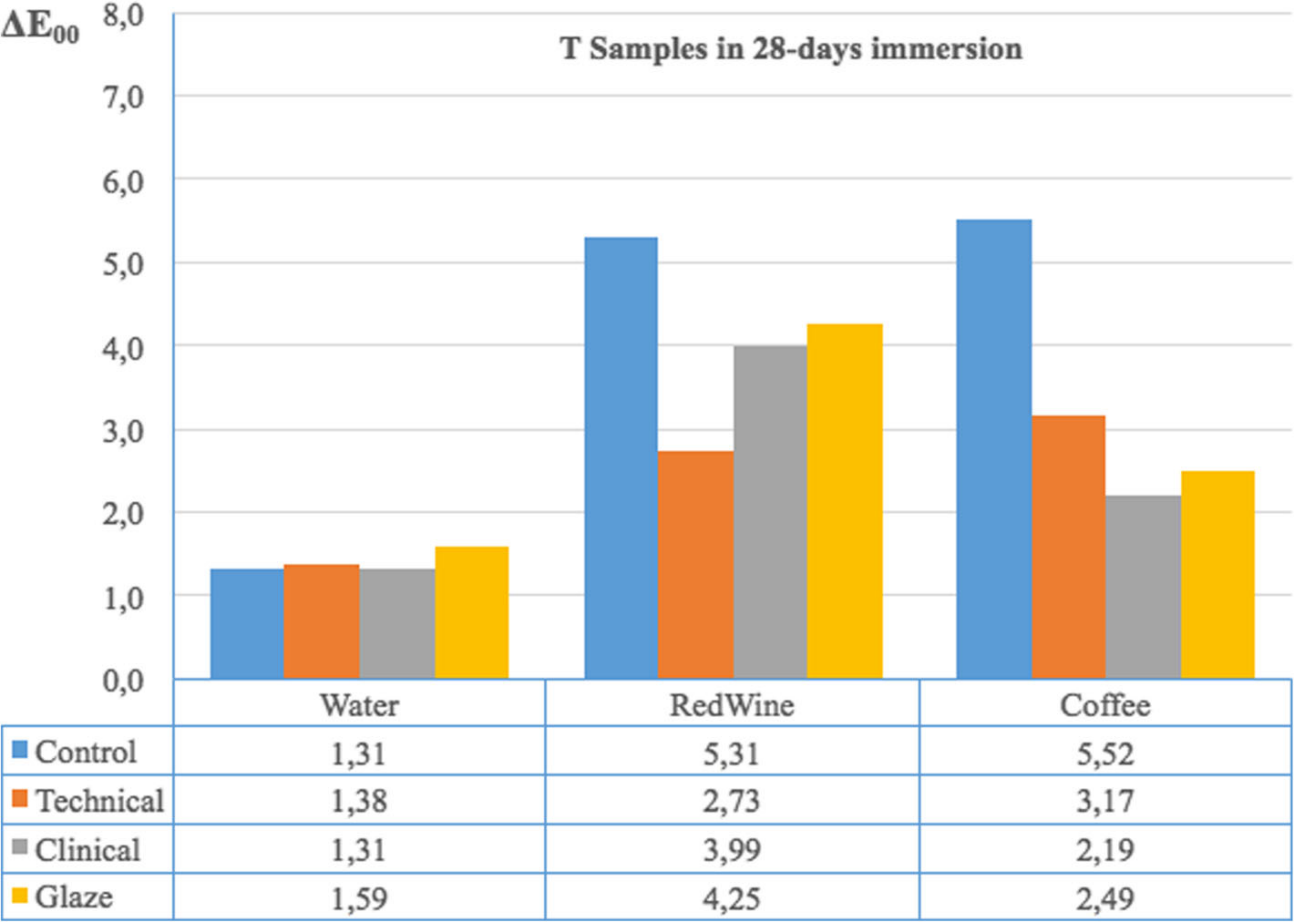
Color changes of translucent polymer infiltrated ceramic network material after 28 days immersion in different drinks

Time dependent discoloration of specimens finished by different procedures applied and kept in distilled water was shown in Table 1. Table 2 shows the time-dependent discoloration of specimens finished with different methods and kept in red wine. According to the results, there was no significant difference between HT Control and HT Glaze groups (*p* > 0.05) but both of these groups showed a statistically significant difference from the HT Technical and HT Clinical groups for 24 h measurements (*p* < 0.05). In both 7 and 28 day measurements, it was observed that the smallest color change occurred in the HT Clinical group and this result was statistically different from the other 3 groups. In 7 and 28 day measurements the smallest color change was observed in the T Technical group which showed a statistically significant difference from the other 3 groups (p < 0.05). Table 3 shows the time-dependent discoloration of specimens finished with different methods and kept in coffee. In 7 and 28 day measurements, it was observed that the greatest color change occurred in the HT Control group and this result was significantly different from the HT Clinical and HT Glaze groups. In 7 and 28 day measurements the greatest color change was observed in the T Control group and it was statistically significantly different from the other 3 groups. In 7 day measurements, the T Technical group showed more color change than the T Clinical and T Glaze groups and this group was statistically significantly different from the T Clinical and T Glaze groups. In 28 day measurements, the T Technical group had more color change than both the T Clinical and T Glaze groups. However, a statistically significant difference was only found between the T Technical and T Clinical groups. Comparing the effectiveness of finishing and polishing systems, Figs. 1 and 2 show the HT and T samples, respectively, immersed for 28 days in different beverages. According to Fig. 1, it can be concluded that the Clinical Kit is the best finishing and polishing system, especially for red wine and distilled water immersion against discoloration for HT samples. Additionally, all finishing and polishing systems were shown to be successful against staining, especially from red wine and coffee immersion (Fig. 2).

**Table 1:** The mean time dependent ΔE00 values and standard deviations calculated by descriptive statistics of the specimens finished with different methods and stored in distilled water

∆E_00_	*HT Control*	*HT Technical*	*HT Clinical*	*HT Glaze*
HT Groups				
24 h	0,71 ± 0,34 ^ab^	0,69 ± 0,14 ^a^	0,52 ± 0,15 ^b, *^	1,53 ± 1,31 ^a^
7 days	0,97 ± 0,39 ^ab^	0,78 ± 0,23 ^a^	1,25 ± 0,21 ^b, *^	1,40 ± 0,97 ^ab^
28 days	0,80 ± 0,38 ^a^	0,51 ± 0,28 ^b, *^	0,36 ± 0,13 ^b, *^	1,67 ± 0,73 ^c^
∆E_00_	*T Control*	*T Technical*	*T Clinical*	*T Glaze*
T Groups				
24 h	0,55 ± 0,14 ^A, *^	0,90 ± 0,21 ^B, *^	0,90 ± 0,22 ^B, *^	0,93 ± 0,43 ^B^
7 days	0,76 ± 0,29 ^A, *^	0,68 ± 0,17 ^A, *^	0,67 ± 0,29 ^A, *^	0,78 ± 0,29 ^A^
28 days	1,31 ± 0,61 ^A, *^	1,38 ± 0,22 ^A, *^	1,31 ± 0,24 ^A, *^	1,59 ± 0,55 ^A, *^

Different superscript let ters indicate statistically significant differences in the same line according to Kruskal-Wallis and Mann-Whitney U tests (*p < 0.05*). Additionally, the group marked with * statistically different from other groups in same column according to Friedman and Wilcoxon tests (*p* < 0.05)

**Table 2:** The mean time dependent ΔE00 values and standard deviations calculated by descriptive statistics of the specimens finished with different methods and stored in red wine

∆E_00_	*HT Control*	*HT Technical*	*HT Clinical*	*HT Glaze*
HT Groups				
24 h	2,75 ± 0,90 ^a, *^	1,59 ± 0,40 ^b^	1,25 ± 0,35 ^b^	2,59 ± 1,09 ^a^
7 days	3,39 ± 0,91 ^a, *^	1,71 ± 0,51 ^b^	1,25 ± 0,34 ^c, *^	3,27 ± 0,64 ^a^
28 days	7,06 ± 1,82 ^a, *^	2,93 ± 0,70 ^b, *^	1,62 ± 0,85 ^c^	5,48 ± 1,25 ^d, *^
∆E_00_	*T Control*	*T Technical*	*T Clinical*	*T Glaze*
T Groups				
24 h	2,77 ± 0,66 ^C^	1,93 ± 0,42 ^A, *^	2,27 ± 0,41 ^B^	2,23 ± 0,39 ^B, *^
7 days	1,99 ± 0,61 ^B^	1,56 ± 0,58 ^A, *^	2,23 ± 0,38 ^B^	3,23 ± 1,04 ^C, *^
28 days	5,31 ± 1,52 ^C, *^	2,73 ± 1,17 ^A, *^	3,99 ± 0,90 ^B, *^	4,25 ± 0,59 ^B, *^

Different superscript letters point out statistically significant differences in same line according to Kruskal-Wallis and Mann-Whitney U (*p* < 0.05). Additionally, the group marked with * statistically different from other groups in same column according to Friedman and Wilcoxon tests (*p* < 0.05)

**Table 3:** The mean time dependent ΔE00 values and standard deviations calculated by descriptive statistics of the specimens finished with different methods and stored in coffee

∆E_00_	*HT Control*	*HT Technical*	*HT Clinical*	*HT Glaze*
HT Groups				
24 h	1,75 ± 0,24 ^a, *^	2,20 ± 0,42 ^b, *^	1,76 ± 0,48 ^ac, *^	1,40 ± 0,47 ^c, *^
7 days	3,68 ± 0,51 ^a^	3,18 ± 1,04 ^ab, *^	2,82 ± 0,82 ^b, *^	2,85 ± 0,67 ^bc^
28 days	4,08 ± 1,06 ^a^	3,62 ± 1,20 ^ab, *^	3,22 ± 0,61 ^b, *^	2,97 ± 1,20 ^b^
∆E_00_	*T Control*	*T Technical*	*T Clinical*	*T Glaze*
T Groups				
24 h	1,33 ± 0,55 ^A, *^	2,45 ± 0,41 ^B, *^	2,18 ± 0,44 ^B^	1,44 ± 0,21 ^A, *^
7 days	5,09 ± 0,91 ^C^	3,56 ± 0,55 ^B^	2,73 ± 0,63 ^A, *^	2,51 ± 0,59 ^A^
28 days	5,52 ± 1,52 ^C^	3,17 ± 1,17 ^B^	2,19 ± 0,52 ^A^	2,49 ± 0,75 ^AB^

Different superscript letters indicate statistically significant differences in the same line according to Kruskal-Wallis and Mann-Whitney U tests (*p* < 0.05). Additionally, the group marked with * statistically different from other groups in same column according to Friedman and Wilcoxon tests (*p* < 0.05)

## Discussion

The initial esthetics and harmony regarding shade of the restorations with natural dentition may be excellent, but the color of dental restorations tends to change over time due to different factors [29]. Successful esthetic prognosis of restorations greatly depends on the maintenance of the initial shade of the restorations after a long time period. Among color-changing factors, the composition and constituents of dental materials have a great importance in the staining resistance of the materials [30], since some of the ingredient materials tend to change the whole shade of the restorations. PICN materials consist of both ceramic and polymer in their structure [30] and thus may have some disadvantages related to discoloration because of polymer chains in their structure or advantages because of the high ceramic content. An important complication of polymers in dentistry is a tendency to staining. It has been shown that surface properties of composites affect the color change and staining [11]. Thus, in the present study both different surface procedures and the effects of staining liquids were evaluated. Regarding the surface finishing procedure at the end of 28 days, among red wine groups, glazed HT specimens showed the greatest color change except for the control group, and HT Clinical specimens showed the smallest color change. In the red wine T groups, the smallest color change was observed in the T Technical group. T Clinical and T Glaze groups had similar color change. In the coffee group at the end of 28 days, the smallest color change was observed in the T Clinical group and it was an acceptable color change. At the end of 28 days, the color change caused by red wine was more prominent than by coffee. The probable reason for the present result may originate from the acidic nature of red wine which may change both the color and surface properties of the composite structure of the material, thus allowing pigments to penetrate readily. Although the staining properties of coffee were more prominent than of red wine, the action mechanisms of the 2 liquids are different because of their pH which may have effects on the composite surfaces.

In previous studies, it was shown that resin matrix composition [4], characterization of filler particles [31], and diet composition affect the degradation of polymers. It has been shown that polymers absorb extrinsic pigments of staining agents because of degradation of their organic matrix [13, 14]. In the present study, red wine and coffee were selected as staining materials. It was shown that the acidic and alcoholic features of red wine cause increased surface roughness of dental materials and alteration in their surface topography and also coffee is the most staining liquid without acidic features [32]. Lawson and Burgess [18] showed that surface polishing conditions have an effect on the stain resistance of CAD/CAM restorative blocks.

Acar et al. [33] investigated the staining of three different polymer-containing materials in coffee with thermocycling. The authors mentioned that the color change of the IPN by coffee was clinically acceptable. In this in vitro study, the effects of different surface procedures, exposure to red wine and coffee and, also translucency in the staining of material were also evaluated. The differences of the present study from the studies of Acar, et al. [33] and Alharbi, et al. [16] are that both HT and T CAD/CAM blocks were included in the study, and different finishing/polishing procedures and the effects of red wine as well as coffee were investigated. Acar, et al. [33] found that the color change for hybrid ceramics was perceivable but acceptable (1.28 < ΔE_00_ < 2.2). According to the results of the study, the different surface procedures have effects on the the staining resistance of CAD/CAM IPN evaluated. It was reported that the clinically acceptable threshold of color difference value for dental ceramics was 2.2 for ΔE_00_ [23]. This indicates that color differences with ΔE_00_ greater than 2.2 were considered as perceivable differences for the observer. The results of the present study reveals that there were statistically significant differences between the HT Technical, HT Clinical and HT Glaze groups kept in red wine for 7 days (*p* < 0.05). However, the color difference was only perceivable in the 7 days Glaze group (ΔE_00_ > 2.2). In the 28 days red wine HT groups, only the HT Clinical group showed a clinically acceptable (non-perceivable) (ΔE_00_ < 2.2) color change. In the T groups kept in red wine, only the T Glaze group showed a clinically perceivable color change (ΔE_00_ > 2.2). In the 28 days measurements, all the T groups showed a clinically perceivable color change (ΔE_00_ > 2.2). Of the T groups kept in coffee for 28 days, it was observed that only the T Clinical group demonstrated a clinically non-perceivable color change (ΔE_00_ < 2.2).

Long-term shade maintenance of tooth-colored restorations is an important factor that has effects o the clinical success of treatment. In the present study one material, 2 different translucency levels, 2 drinks and different finishing/polishing procedures were evaluated. It is also accepted that the exposure time to drinks is an important factor for the discoloration of dental materials. Thus, different exposure periods to drinks were also investigated in the present study. In a previous study, it has been shown that 24 h exposure to drinks in vitro corresponds to 1 month in vivo [34]. In this study, the exposure periods to the staining solutions correspond to 1, 7 and 28 months respectively. Knowledge of the staining resistance of a restoration material may be helpful to the clinician when selecting the material and its translucency in clinical practice. The above-mentioned results on the effects of coffee and red wine consumption may also help the clinician in the decision-making process of dental material selection. However, the present study evaluated an IPN material in in vitro conditions. Further in vitro and in vivo studies are needed to determine the effects of staining factors on the new IPN materials.

## Conclusions

Within the limitations of this study, it can be concluded that various surface procedures for finishing and also translucency levels of an IPN have effects on staining resistance since polished samples showed a smaller color change than the unpolished groups. In particular, technical and clinical polishing systems should be preferred for the clinical to final stage of IPN restorations.

## Abbreviations

CAD/CAM: Computer aiding design/Computer aiding manufacting
CIELAB: Commission Internationale de l’Eclairage
HT: High translucent
IPN: Interpenetrating network composite
PICN: Polymer-infiltrated ceramic network
T: Translucent
